# Implementing a streamlined data access governance process for the release of routinely collected electronic health record data for research

**DOI:** 10.23889/ijpds.v9i5.2809

**Published:** 2024-09-18

**Authors:** David Clark

**Affiliations:** 1 Public Health Scotland

## Objectives

In Australia, there are no standardised processes for the release of Electronic Health Record (EHR) data for research. We describe our streamlined ethics and data access governance framework for the release of EHR data for research.

## Approach

In consultation with our academic and health service partner organisations and with reference to national and international standards, we developed a process to safely and efficiently release EHR data for research. The Five Safes Framework guided our processes ensuring: safe people; safe projects; safe settings; safe data; and safe outputs. Piloting of processes has commenced.

## Results

Streamlined data access and release processes acceptable to all stakeholders were implemented using an online system. Independent researcher and consumer review of an Expression of Interest, followed by ethics review/approval from the health service research office ensures projects are methodologically and ethically sound. Completion of a detailed technical assessment form, implementation of a signed data use agreement and release of de-identified data to a secure e-research environment promotes safe data and settings. Constraints around publication are enforced via pre-publication review ensuring safe outputs. Nine projects are in process using our system with an average of 4 days for EOI review and 6 days for TAF review.

## Conclusion

We have established a streamlined data access pathway to minimise duplication of effort and allow timely and secure access to EHR research data. We have built in efficiencies and promoted a cultural shift for clinicians who have previously not had data access processes governed in this way.

